# Motion artifacts occurring at the lung/diaphragm interface using 4D CT attenuation correction of 4D PET scans

**DOI:** 10.1120/jacmp.v12i4.3502

**Published:** 2011-11-15

**Authors:** Joseph H. Killoran, Victor H Gerbaudo, Marcelo Mamede, Dan Ionascu, Sang‐June Park, Ross Berbeco

**Affiliations:** ^1^ Department of Radiation Oncology Dana‐Farber/Brigham & Women's Cancer Center Boston; ^2^ Division of Nuclear Medicine, Department of Radiology Brigham and Women's Hospital, Harvard Medical School Boston MA

**Keywords:** 4D PET, motion artifacts, attenuation correction

## Abstract

For PET/CT, fast CT acquisition time can lead to errors in attenuation correction, particularly at the lung/diaphragm interface. Gated 4D PET can reduce motion artifacts, though residual artifacts may persist depending on the CT dataset used for attenuation correction. We performed phantom studies to evaluate 4D PET images of targets near a density interface using three different methods for attenuation correction: a single 3D CT (3D CTAC), an averaged 4D CT (CINE CTAC), and a fully phase matched 4D CT (4D CTAC). A phantom was designed with two density regions corresponding to diaphragm and lung. An 8 mL sphere phantom loaded with 18F‐FDG was used to represent a lung tumor and background FDG included at an 8:1 ratio. Motion patterns of sin(x) and $\sin^{4}(x)$ were used for dynamic studies. Image data was acquired using a GE Discovery DVCT‐PET/CT scanner. Attenuation correction methods were compared based on normalized recovery coefficient (NRC), as well as a novel quantity “fixed activity volume” (FAV) introduced in our report. Image metrics were compared to those determined from a 3D PET scan with no motion present (3D STATIC). Values of FAV and NRC showed significant variation over the motion cycle when corrected by 3D CTAC images. 4D CTAC‐ and CINE CTAC–corrected PET images reduced these motion artifacts. The amount of artifact reduction is greater when the target is surrounded by lower density material and when motion was based on $\sin^{4}(x)$. 4D CTAC reduced artifacts more than CINE CTAC for most scenarios. For a target surrounded by water equivalent material, there was no advantage to 4D CTAC over CINE CTAC when using the sin(x) motion pattern. Attenuation correction using both 4D CTAC or CINE CTAC can reduce motion artifacts in regions that include a tissue interface such as the lung/diaphragm border. 4D CTAC is more effective than CINE CTAC at reducing artifacts in some, but not all, scenarios.

PACS numbers: 87.57.qp, 87.57.cp

## I. INTRODUCTION

Computed tomography (CT) and positron emission tomography (PET)^†^ are imaging modalities of different qualities which are complementary. The development of PET/CT scanners has allowed for both modalities to be acquired in a single session with the significant advantage that their spatial coordinates are inherently linked. In addition to providing important diagnostic information, the CT component of PET/CT also provides information necessary for attenuation correction (CTAC) of the emission data during PET image reconstruction. This step is essential for the PET image data to be accurate for quantitative applications such as standardized update value (SUV) calculation.

Both PET and PET/CT imaging have multiple clinical applications. In radiotherapy, PET is frequently used to assist physicians in defining target volumes to be irradiated.^(^ ^1^ ^)^ Since its inception, numerous researchers have evaluated the potential for PET and PET/CT to be employed in the treatment planning process for lung cancer, with a number of studies focusing specifically on the impact of PET on target volume definition.^(^ ^2^ ^–^ ^5^ ^)^ Understanding the efficacy of PET in this context is hindered by the lack of a suitable ground truth, and the ideal methodology for quantitative use of PET or PET/CT in these processes remains a subject of debate.^(^ ^6^ ^,^ ^7^ ^)^ It has been shown, however, that interobserver variability is reduced when PET/CT is used for lung cancer radiotherapy target definition.^(^ ^2^ ^)^ In any case, a quantitative application of PET images clearly requires that the image data possess quantitative accuracy.

While applications of PET to lung cancer are well established, this anatomical site in particular presents a well‐known challenge due to internal respiratory motion. Motion produces two distinct challenges for PET/CT. First, it blurs PET emission data and reduces SUVs. In addition, the fast acquisition times of CT produce images which represent a specific moment within the respiratory cycle, while the much slower acquisition time of PET produces an image that represents the average of many respiratory cycles. This mismatch in phases can produce artifacts and affect SUV values.^(^ ^8^ ^–^ ^12^ ^)^ This is particularly true when the region of interest is at a boundary between tissue types of different densities, most significantly, at the lung/diaphragm interface.^(^ ^11^ ^,^ ^13^ ^,^ ^14^ ^)^ It has been reported that using CT data acquired at deep inspiration can produce severe artifacts,^(^ ^15^ ^)^ and also that CT protocols using normal expiration breath‐hold or partial breath‐hold acquisition can reduce artifacts in this region.^(^ ^15^ ^,^ ^16^ ^)^ Others have proposed that errors due to attenuation correction can be reduced by using a Cine‐CT to produce an averaged (or max) image dataset for correction purposes.^(^ ^17^ ^–^ ^20^ ^)^

**Figure 1:**
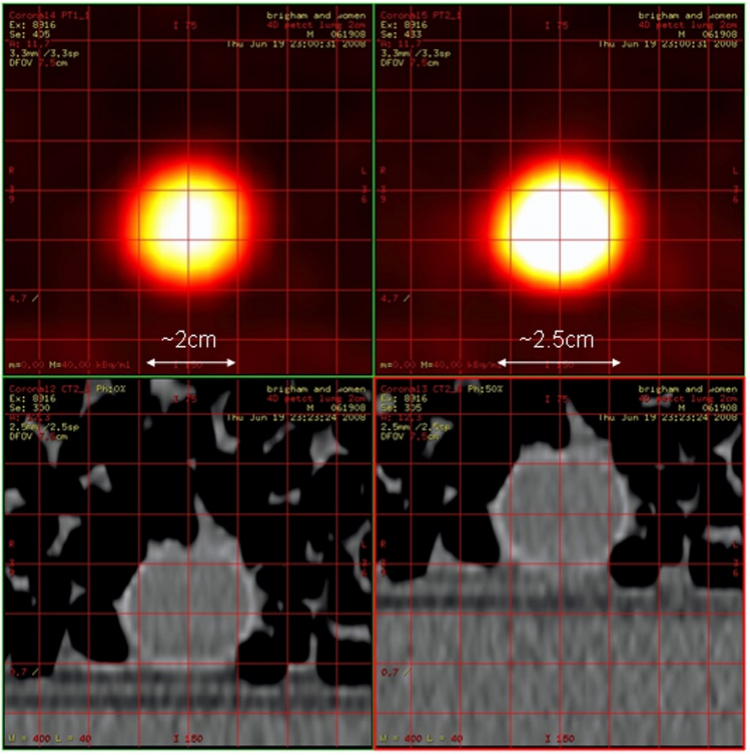
Illustration of the type of artifact that can occur from using a single CT image for attenuation correction when motion is present. Both PET images are reconstructed from the same raw data of the 0% phase bin of a 4D gated PET acquisition. The PET image on the left has been corrected for attenuation using CT 0% phase CT data (phase‐matched), while the image on the right has been corrected using CT data from the 50% phase (phase‐mismatched) resulting in overcorrection.

Gated PET (4D PET) can be used to reduce motion artifacts due to the blurring of the emission data and the reconstructed images. However, a 4D PET study may or may not be corrected for attenuation by an analogously acquired phase matched 4D CT scan (4D CTAC). Figure 1 illustrates the imaging artifact that can occur when a 4D PET image is corrected using CTAC data which is out of phase of the 4D PET data. While it has been reported that it is feasible to minimize motion artifacts by using 4D PET corrected by 4D CT in patients in a clinical setting,^(^ ^21^ ^–^ ^23^ ^)^ the addition of a 4D CT adds considerable complexity, as well as increased patient dose. As a consequence, clinical centers may avoid these complications by using 4D PET imaging protocols with attenuation correction based on 3D CT. A number of studies have compared different methods for 4D PET attenuation correction in patients^(^ ^21^ ^)^ and phantoms,^(^ ^24^ ^–^ ^26^ ^)^ as well as simulated data based on 3D PET and 4D CT in patients.^(^ ^13^ ^)^ As expected, these studies conclude generally that 4D PET reduces motion artifacts, and that 4D PET corrected by phase matched 4D CT is the most quantitatively accurate approach. However, 4D CT may not always be appropriate or available in the clinical setting due to scanner capabilities and/or the additional patient dose and complexity it adds to the scan. For this reason, it is important to fully understand the relative benefits of different attenuation correction methods.

The purpose of this study is to evaluate the performance of 4D PET imaging, and examine the choice of attenuation correction method using a phantom designed to represent the lung–diaphragm interface. While it is well‐known that this interface is a source of attenuation correction errors, we know of no other report that has employed a phantom specifically designed to model this anatomical region. In particular, we sought to quantify motion artifacts that occur during the attenuation correction portion of PET image reconstruction.

## II. MATERIALS AND METHODS

Phantom studies were specifically designed to evaluate 4D PET images of targets near a density interface using three different methods for attenuation correction: a single 3D CT (3D CTAC), an averaged 4D CT (CINE CTAC), and a fully phase matched 4D CT (4D CTAC).

### A.1 Phantom

**Figure 2:**
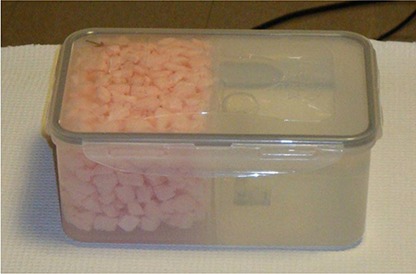
The phantom used for our studies. It consists of a watertight plastic box with two sections designed to represent lung and liver.

**Figure 3:**
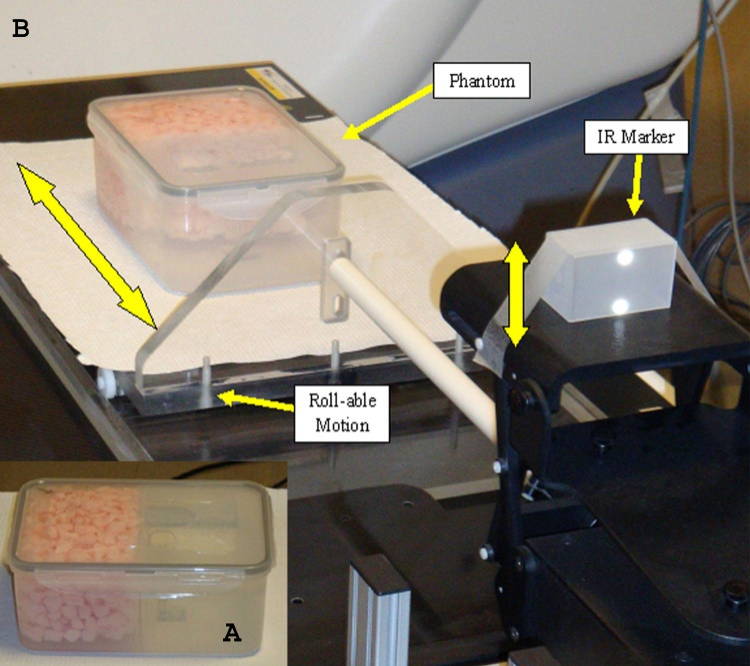
The phantom positioned for scanning using the dynamic phantom controller.

The phantom was designed to approximate the geometry of the anatomical region between the lung and diaphragm. A watertight plastic box was used which consisted of two sections: a low density section to represent lung and a water‐filled section to approximate liver. The “lung” side was filled with a mixture of water and Styrofoam packing material (‘peanuts’), as shown in Fig. 2. CT images of the phantom show an average HU value of −630 on the ‘lung’ side and close to zero on the ‘liver’ side, being representative of the relative densities at the lung/ diaphragm interface. A small amount of 18F‐FDG (~300 μCi) was added to both sections to represent background activity. The tumor was represented by an 8 mL sphere phantom (Hollow Sphere Set, Model ECT/HS/SET6, Data Spectrum Corporation, Hillsborough, NC) loaded with 18F‐FDG which could be placed on either side of the border. The phantom was situated on a small platform with wheels, and the entire assembly was connected to a commercially available dynamic phantom (Dynamic Thorax Phantom, Computerized Imaging Reference Systems (CIRS), Norfolk, VA) which was driven by a computerized motion controller. Figure 3 shows a photo of the phantom in position for scanning.

### A.2 PET/CT scanner

A GE PET/CT scanner (Discovery DVCT‐PET/CT, GE Medical Systems, Milwaukee, WI) was used for these studies. The PET scanner has a 15 cm axial field of view which is combined with a 64 slice CT scanner. The Varian RPM motion tracking system (Real‐time Position Management System, Varian Medical Systems, Palo Alto, CA) is used for motion tracking based on an IR reflective maker. Clinically this marker is normally placed on the anterior surface of the patient's abdomen to allow respiratory motion monitoring via an optical camera. For this phantom study, the marker box was placed on a small platform which moves in the AP direction synchronously with the phantom's SI motion.

### A.3 Imaging studies: acquisition

CT scans for attenuation correction were acquired using a standard protocol: a 0.5 s rotation time, 32×1.25 mm scan acquisition reconstructed to 3.75 mm thick slices with a 500 mm axial field of view. Tube potential of 140 kVp and current of 75 mAs were used. Traditional 3D PET/ CT studies were acquired both with and without the phantom in motion. 4D PET scans were acquired with the phantom in motion while the RPM system simultaneously recorded the motion of the IR reflective marker. The motion trace and images from the CINE scan were then transferred to a GE workstation (Advantage 4D, Advantage Windows, GE Medical Systems, Milwaukee, WI) for reconstruction of a phase binned 4D image dataset using the Advantage 4D application. These data were then transferred back to the PET/CT console to be used for attenuation correction of PET emission data.

10 μCi of 18F‐FDG, a concentration of (1.25 μCi/cc), was loaded into the sphere phantom “target”, as well as a lower concentration (300 μCi−0.16 μCi/cc) into both phantom compartments to provide an approximate target to background ratio of 8:1. A series of scans was performed with the target vial on the “liver” side of the phantom and then repeated with the vial placed on the “lung” side. The vial was reloaded with 18F‐FDG prior to the second series.

Data was acquired with the phantom moving according to two different motion patterns, a simple sinusoid sin(x) as well as $\sin^{4}(x)$. The simple sinusoid sin(x) provides a baseline for motion effects but may not be a realistic model of actual breathing patterns. The motion pattern $\sin^{4}(x)$, as suggested by Lujan et al. and Seppenwoolde et al.,^(^ ^27^ ^,^ ^28^ ^)^ more closely represents an actual realistic breathing motion than the simple sinusoid. For both motion patterns an amplitude of 2 cm over a period of 4.5 sec was used with the direction of motion towards and away from the gantry, corresponding to the superior/inferior direction in a patient.

Three basic types of imaging studies were performed:

- 3D Static: To set a baseline for comparison, a traditional 3D PET/CT scan was first acquired without phantom motion. The PET portion was performed using a 2 min acquisition time.
- 3D Dynamic: With the phantom in motion, a 3D PET/CT scan was acquired using the same parameters as for 3D Static, for both phantom motion patterns sin(x) and $\sin^{4}(x)$.
- 4D Dynamic: Next, a gated study was acquired consisting of a 4D CT scan and a 20 min PET acquisition. 4D CT and 4D PET scans were acquired with the phantom in motion while the RPM system simultaneously recorded the motion of the IR reflective marker. CT data was processed into a 10‐bin 4D image set using GE Advantage 4D software.

The 3D Static and 3D Dynamic studies were both acquired using a 2 min acquisition time in order to match the average acquisition time per bin of the 4D studies (10 bins over 20 minutes acquisition). Since a 4 min acquisition time is more typical for clinical 3D PET, both 3D Static and 3D Dynamic studies were repeated using a 4 min acquisition. A comparison of the 2 min vs. 4 min acquisition images revealed only a small effect of the increased acquisition time (a difference of ~2% or less for maximum and average activity) for our phantom. Thus, for our subsequent analysis comparing 3D to 4D image data, the 2 min acquisition images were used exclusively.

### A.4 Imaging studies: attenuation correction

PET images were reconstructed, based on an existing clinical protocol, using a 128 × 128 matrix and an iterative ordered subsets expectation maximization (OSEM) algorithm (2 iterations, 30 subsets), yielding a volume of 47 sections with thickness and spacing of 3.27 mm and an in‐plane pixel size of 4.69×4.69 mm.

#### A.4.1 3D image studies (static and dynamic)

Examples of both the 3D Static and 3D Dynamic scans were reconstructed using a single 3D CTAC dataset. This represented the current typical practice and was a basis for comparison. For the 3D Dynamic study, an ungated PET scan of a moving target was corrected with a 3D CTAC that was started at a random breathing phase. Since this example was meant to represent clinical practice, we did not make any effort to determine or control the phase. The result, therefore, represented one example from a random distribution. We did not feel that it was necessary to explore the range of possible outcomes for the 3D Dynamic case as this was achieved by extracting specific extremes of motion from the 4D studies.

#### A.4.2 4D image studies

The 4D PET studies were reconstructed into 10‐bin 4D images using the PET/CT dedicated computer console. The uncorrected 4D PET images were then corrected for attenuation using the four different attenuation correction scenarios described below.

#### A.4.3 3D CTAC

A single 3D CTAC image set was used for attenuation correction of the 4D PET datasets. This was closest to the current clinical practice, in which the fast CT acquisition speed will capture the patient (or phantom) at a random phase within the respiratory cycle. To determine the range of possible outcomes from using a single 3D CTAC image set, the 4D PET reconstruction was also performed with single phases picked from a 4D CT dataset, capturing the extremes of motion. These are described in more detail below.

- 3D‐CTAC‐00: the 0% phase CT images extracted from the 4D CT; this is analogous to using a breath hold ‘inhale’ CT scan
- 3D‐CTAC‐50: the 50% phase CT images extracted from the 4D CT; this is analogous to a breath‐hold ‘exhale’ CT scan

It should be emphasized that CTAC‐00 and CTAC‐50 are not practical methods for attenuation correction per se. By analysis of the images reconstructed using these extremes, the range of possible outcomes can be determined from the 3D‐CTAC.

#### A.4.4 CINE CTAC

To reduce artifacts associated with using a single “snap‐shot” CT scan, a CINE CTAC scan was used for attenuation correction. Using CINE mode, the scanner acquires multiple images at each couch position. When used for attenuation correction, the images are combined by the console reconstruction software to generate a correction based on the average density at each image location.

#### A.4.5 4D CTAC

A 4D CT image set was acquired for attenuation correction and phase matched to the 4D PET data. In this way, each bin of the 4D PET data was corrected using CT data of the same nominal phase.

### A.5 Analysis

A computer program was developed, using the Java programming language, to read DICOM image data files and calculate quantitative measures for comparison of the different images. Values for activity/mL for each voxel were corrected for decay using the 3D Static image study as the basis for comparison. Two specific quantities are presented for analysis.

- Normalized Recovery Coefficient (NRC): The maximum value of activity/mL, represented as a percentage relative to the maximum value found for the corresponding 3D Static image acquisition. The presence of motion reduces this value.^(^ ^24^ ^)^ A 4D imaging technique can be evaluated in terms of its ability to increase NRC towards the levels observed when no motion is present.

While the recovery coefficient is commonly used in the analysis of PET images, it represents a single voxel (the max) and is disproportionately affected by image noise. To address these deficiencies we devised a quantity more directly related to the ability of the specific correction method to quantify the true volume of the target vial. We call this new quantity the *Fixed Activity Volume* (FAV).

The FAV was determined by first making an array ‘list’ of the voxels in the image set sorted in descending order by activity/mL. From this list, the total amount of activity was found representing the known volume of the vial (8 mL) for the 3D STATIC image set. With this total activity value as a reference (corrected for decay), the amount of volume, in mL, needed to find the same amount of total activity in the dynamic image studies was calculated. The presence of motion spreads the activity over a large area and increases the FAV. The 4D imaging techniques can be compared in terms of how well they lower FAV values towards those observed without motion. We present FAV values as a percentage of the known volume (8 mL) of the target vial.

Values for FAV and NRC were calculated for each of the 3D Static, 3D Dynamic, and the 4D Dynamic studies reconstructed with the methods of attenuation correction described above. For the 3D cases, only one value per image set was produced. For the 4D studies, a value was determined for each phase of the image dataset, producing a range of possible values over the motion cycle.

## III. RESULTS

Results for NRC and FAV are summarized in Tables 1 and 2, respectively. Values are tabulated for the average, standard deviation, minimum, maximum, and range of values for FAV and NRC. For each quantity (FAV or NRC), values are shown for data collected with the vial on the ‘lung side’ and on the ‘liver side’ using motion patterns sin(x) and $\sin^{4}(x)$. Both FAV and NRC are normalized so that 100% represents the baseline: the 3D Static' dataset. For the values of FAV, 100% also represents the known volume of the target vial (8 mL).

**Table 1:** Summary of the results comparing the different attenuation correction methods in terms of normalized recovery coefficient (NRC). These values are normalized such that 100% represents the baseline recovery for the 3D Static dataset. The average, standard deviation, minimum, maximum, and range were determined over all the phases of a 10‐bin 4D PET image dataset reconstructed using the attenuation correction method in question. Key values are highlighted to illustrate the differences in the range of values for the different attenuation correction methods.

*Normalized Recovery Coefficient*										
		*Sin(x) in Lung*					$Sin^{4}(x)$ *in Lung*			
	*Average*	*STD*	*Min*	*Max*	*Range*	*Average*	*STD*	*Min*	*Max*	*Range*
CTAC 00	87.0	7.0	75.5	93.6	18.1	87.2	7.9	75.3	97.9	22.6
CTAC 50	112.5	15.4	91.8	131.1	39.4	110.5	16.2	92.5	136.9	44.4
3D CTAC	99.8	**17.5**	75.5	131.1	**55.7**	98.9	**17.2**	75.3	136.9	**61.6**
4D CINE	97.3	**8.4**	85.5	109.1	**23.6**	101.0	**11.7**	87.0	123.0	**36.0**
4D CTAC	94.3	**2.6**	89.0	97.7	**8.7**	96.8	**3.4**	91.8	102.5	**10.7**

		*Sin(x) in Lung*					$Sin^{4}(x)$ *in Lung*			
	*Average*	*STD*	*Min*	*Max*	*Range*	*Average*	*STD*	*Min*	*Max*	*Range*
CTAC 00	92.7	7.4	75.8	100.9	25.1	92.3	9.5	76.1	102.6	26.5
CTAC 50	97.8	5.5	88.0	108.7	20.6	99.0	4.5	93.2	108.5	15.3
3D CTAC	95.3	**6.9**	75.8	108.7	**32.8**	95.7	**8.0**	76.1	108.5	**32.4**
4D CINE	95.6	**5.3**	88.1	105.8	**17.8**	96.5	**6.6**	84.7	106.0	**21.4**
4D CTAC	97.6	**5.5**	87.9	108.7	**20.7**	99.1	**4.8**	92.3	109.0	**16.7**

**Table 2:** Summary of the results comparing different methods for attenuation correction in terms of fixed activity volume (FAV). FAV values are normalized to the known volume of the sphere phantom (8 ml). The average, standard deviation, minimum, maximum, and range were determined over all the phases of a 10‐bin 4D PET image dataset reconstructed using the attenuation correction method in question. Key values are highlighted to illustrate the differences in the range of values for the different attenuation correction methods.

*Fixed Activity Volume*										
		*Sin(x) in Lung*					$Sin^{4}(x)$ *in Lung*			
	*Average*	*STD*	*Min*	*Max*	*Range*	*Average*	*STD*	*Min*	*Max*	*Range*
CTAC 00	137.8	21.2	112.6	166.7	54.1	144.2	22.3	114.4	175.7	61.3
CTAC 50	91.1	18.1	69.4	114.4	45	96.5	18	71.2	118	46.8
3D CTAC	114.5	**30.7**	69.4	166.7	**97.3**	120.4	**31.4**	71.2	175.7	**104.5**
4D CINE	111.7	**17**	90.1	134.2	**44.1**	111.1	**18**	84.7	134.2	**49.5**
4D CTAC	115.2	**5.9**	107.2	126.1	**18.9**	115.5	**9.5**	100.9	130.6	**29.7**

		*Sin(x) in Lung*					$Sin^{4}(x)$ *in Lung*			
	*Average*	*STD*	*Min*	*Max*	*Range*	*Average*	*STD*	*Min*	*Max*	*Range*
CTAC 00	128.8	21	108.2	168.2	60	136.9	26	107.3	172.7	65.5
CTAC 50	111.8	5.8	99.1	118.2	19.1	111.9	3.8	107.3	117.3	10
3D CTAC	120.3	**17.3**	99.1	168.2	**69.1**	124.4	**22.2**	107.3	172.7	**65.5**
4D CINE	117.1	**8.9**	105.5	134.5	**29.1**	118.9	**9.5**	107.3	133.6	**26.4**
4D CTAC	113.1	**6.6**	99.1	121.8	**22.7**	112.5	**4.6**	107.3	120.9	**13.6**

**Figure 4:**
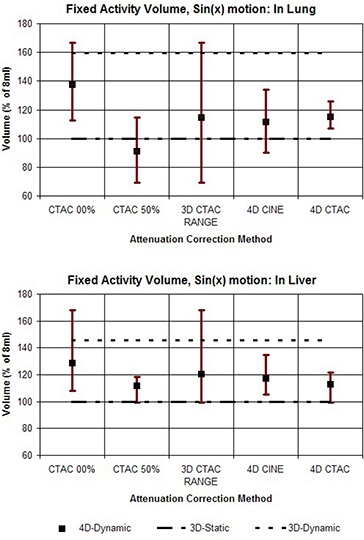
Graphs comparing the different methods for attenuation correction based on the volume of voxels determined from PET images needed to recover the known amount of activity (Fixed Activity Volume). For each method, the value for FAV was calculated for each of the 10 phases of the 4D PET image sets and the results are shown as a range of values. The upper graph shows the results for the target vial on the ‘lung’ side of the phantom, and the lower graph shows the results for the vial on the ‘liver’ side. The phantom motion was according to sin(x).

**Figure 5:**
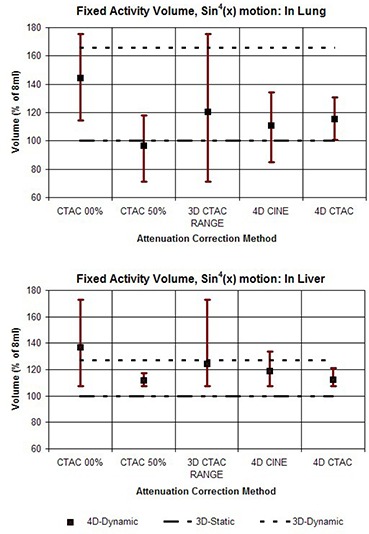
Graphs comparing the different methods for attenuation correction based on the volume of voxels determined from PET images needed to recover the known amount of activity (Fixed Activity Volume). For each method, the value for FAV was calculated for each of the 10 phases of the 4D PET image set and the results are shown as a range of values. The upper graph shows the results for the target vial on the ‘lung’ side of the phantom and the lower graph shows the results for the vial on the ‘liver’ side. The phantom motion was according to sin(x) raised to the 4th power.

**Figure 6:**
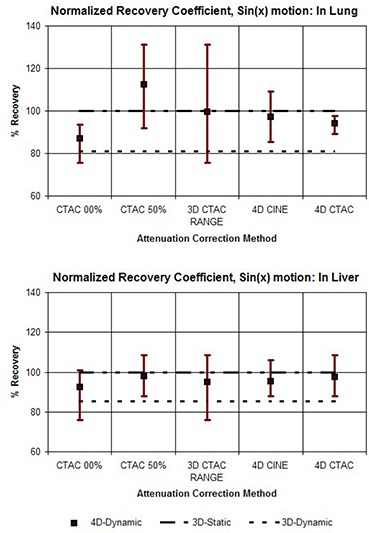
Graphs comparing the different methods for attenuation correction based on the normalized recovery coefficient. For each method, the value for NRC was calculated for each of the 10 phases of the 4D PET image set and the results are shown as a range of values. The upper graph shows the results for the target vial on the ‘lung’ side of the phantom and the lower graph shows the results for the vial on the ‘liver’ side. The phantom motion was according to sin(x).

**Figure 7:**
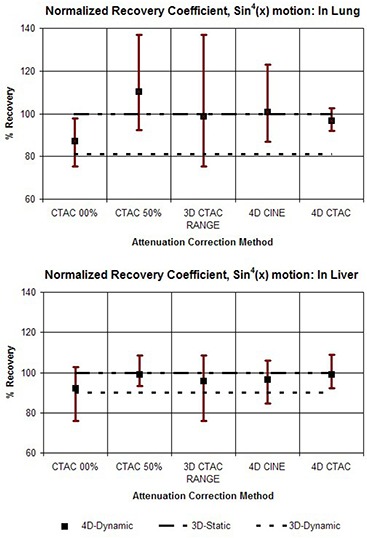
Graphs comparing the different methods for attenuation correction based on the normalized recovery coefficient. For each method, the value for NRC was calculated for each of the 10 phases of the 4D PET image set and the results are shown as a range of values. The upper graph shows the results for the target vial on the ‘lung’ side of the phantom and the lower graph shows the results for the vial on the ‘liver’ side. The phantom motion was according to sin(x) raised to the 4th power.

In addition, the results are shown in graphs which compare the various attenuation correction methods in terms of the average values for comparison, as well as the range of values from each of the 10 phases of the motion cycle. Values representing the 3D Static and 3D Dynamic cases are shown for comparison purposes as flat lines. The graphs shown in Fig. 4 compare the range of outcomes of FAV for different correction methods for the case of the vial on the corresponding ‘lung’ and ‘liver’ sides of the 4D phantom when a motion pattern of sin(x) is used. The heading “3D CTAC RANGE” combines data from the extreme phases, 0% and 50%. This illustrates the full range of outcomes that can be produced when a single 3D CTAC image set of an essentially random phase is used. Figure 5 shows the results for FAV for the phantom motion pattern of $\sin^{4}(x)$. The values for normalized recovery coefficient for sin(x) motion are shown in Fig. 6, while values for $\sin^{4}(x)$ motion are shown in Fig. 7.

## IV. DISCUSSION

To assess the results presented in Figs. 4–7, it is helpful to keep in mind that the primary purpose of 4D imaging is to reduce or eliminate motion artifacts. As such, the effectiveness of a specific 4D imaging and reconstruction method can be evaluated in terms of its ability to effectively stop motion. Within the context of these phantom studies the 3D Static case, in which no motion is present, serves as a useful benchmark for comparison. By contrast, the 3D Dynamic values represent an example of the other extreme — motion is present but not accounted for.

What is immediately apparent from the results is that the use of a 3D CTAC, with no control over the specific phase it represents, can produce a wide range of results when motion is present. Both NRC and FAV metrics show that the 4D correction methods produce a clear reduction in the range of variation, reducing the effects of motion when compared to 3D CTAC. Between the two 4D techniques, 4D CTAC was generally more effective than 4D CINE at reducing motion effects, particularly when the target was placed on the low density (lung) side of the phantom. With the target on the “liver” side, the difference between 4D CINE and 4D CTAC was reduced. In particular, for the “liver” side studies, the 4D CTAC method produced a somewhat larger amount of variation in NRC than the 4D CINE method when the sin(x) motion pattern was used. This was not the case for the $\sin^{4}(x)$ pattern, however, for which the 4D CTAC did have an advantage over 4D CINE for scans on the “liver” side.

Our results also illustrate that the errors due to incorrect density correction as a result of motion are distinctly different if the target is on the “liver” side or the “lung” side. For a target region on the “lung” side, depending on the specific phase of the CTAC, an error in the density correction can occur in two distinct ways: either by low density “lung” without the target present (undercorrection), or by higher density “liver” (overcorrection). A target on the “liver” side, however, is surrounded by a material of similar density. Motion due to inspiration may result in an undercorrection if lung is substituted for liver, but motion in the other direction will simply substitute more liver in place of target plus liver, resulting in virtually no error in the correction. As such, a target in this location is generally only subject to undercorrection and not overcorrection, while a target on the “lung” side suffers from both.

As a consequence of this, when the target is on the higher density “liver” side, a correction based on the CTAC 50% appears to do about the same, or even slightly better, than the 4D CTAC. This does suggest that a strategy of using end expiration 3D CT for correction could be a reasonable alternative to either 4D CINE or 4D CTAC in some situations. However, this approach would require that the target location be reliably known a priori, which will not generally be the case in clinical situations.

While our results show that 4D CT correction methods will reduce the effects of phase mismatch between PET emission and CTAC data, a number of factors complicate the use of 4D CT in a clinical setting. Whereas the addition of a 4D CT series by itself does not add significantly to the acquisition time of a 4D PET study, 4D PET acquisition requires a substantially longer acquisition time. While the acquisition of 4D CT data (either CINE or 4D CTAC) does not add significantly to the total acquisition time, it is important to consider that 4D studies involve substantially increased radiation dose to the patient. The current method used for our study for 4D PET image reconstruction using 4D CTAC also requires significant additional effort on the part of the scanner operator for two specific reasons. The 4D CT data must be phase‐binned using a separate imaging workstation and transferred back to the scanner console and, once transferred, must be manually matched to the appropriate phase of the uncorrected 4D PET. As such, in current form, the 4D CTAC method does not appear to be a practical method for routine clinical use. This is not the case for the 4D CINE method, for which the only drawback is the additional radiation exposure to the patient.

## V. CONCLUSIONS

Based on phantom studies using a simulated lung/diaphragm interface, 4D PET images reconstructed using both the 4D CINE and 4D CTAC attenuation correction methods showed fewer motion artifacts when compared to 4D PET images reconstructed using a traditional 3D CTAC. A larger effect was observed when the target was in low‐density material (simulated lung) as opposed to water equivalent (simulated liver) material, and also when motion was according to $\sin^{4}(x)$ rather than sin(x). The 4D CTAC correction method was more effective at reducing artifacts than the 4D CINE method for most scenarios, with the exception of a target in high‐density (liver) material and a motion pattern of sin(x) for which the two methods were approximately equivalent. We conclude that when 4D PET images are acquired near a tissue interface such as the lung/diaphragm border, 4D attenuation correction techniques are of value for reducing motion artifacts.

## Footnotes

† Throughout this report, references to positron emission tomography and/or PET imaging refer to PET using the radiotracer FDG.

## References

[1] MacManus M , Nestle U , Rosenzweig KE , et al. Use of PET and PET/CT for radiation therapy planning: IAEA expert report 2006–2007. Radiother Oncol. 2009;91(1):85–94. 19100641 10.1016/j.radonc.2008.11.008

[2] Ashamalla H , Rafla S , Parikh K , et al. The contribution of integrated PET/CT to the evolving definition of treatment volumes in radiation treatment planning in lung cancer. Int J Radiat Oncol Biol Phys. 2005;63(4):1016–23. 15979817 10.1016/j.ijrobp.2005.04.021

[3] Faria SL , Menard S , Devic S , et al. Impact of FDG‐PET/CT on radiotherapy volume delineation in non‐small‐cell lung cancer and correlation of imaging stage with pathologic findings. Int J Radiat Oncol Biol Phys. 2008;70(4):1035–38. 17996383 10.1016/j.ijrobp.2007.07.2379

[4] Nestle U , Kremp S , Grosu AL . Practical integration of [18F]‐FDG‐PET and PET‐CT in the planning of radiotherapy for non‐small cell lung cancer (NSCLC): the technical basis, ICRU‐target volumes, problems, perspectives. Radiother Oncol. 2006;81(2):209–25. 17064802 10.1016/j.radonc.2006.09.011

[5] Bradley J , Thorstad WL , Mutic S , et al. Impact of FDG‐PET on radiation therapy volume delineation in non‐small‐cell lung cancer. Int J Radiat Oncol Biol Phys. 2004;59(1):78–86. 15093902 10.1016/j.ijrobp.2003.10.044

[6] Biehl KJ , Kong FM , Dehdashti F , et al. 18F‐FDG PET definition of gross tumor volume for radiotherapy of non‐small cell lung cancer: is a single standardized uptake value threshold approach appropriate? J Nucl Med. 2006;47(11):1808–12. 17079814

[7] Wong CY , Mahajan P , Yan D . Dynamic threshold for radiation target volume by PET/CT. J Nucl Med. 2007;48(5):849; author reply 847. 17475975 10.2967/jnumed.106.038141

[8] Cohade C , Osman M , Marshall LN , Wahl RN . PET‐CT: accuracy of PET and CT spatial registration of lung lesions. Eur J Nucl Med Mol Imaging. 2003;30(5):721–26. 12612809 10.1007/s00259-002-1055-3

[9] Osman MM , Cohade C , Nakamoto Y , Wahl RL . Respiratory motion artifacts on PET emission images obtained using CT attenuation correction on PET‐CT. Eur J Nucl Med Mol Imaging. 2003;30(4):603–06. 12536242 10.1007/s00259-002-1024-x

[10] Erdi YE , Nehmeh SA , Pan T , et al. The CT motion quantitation of lung lesions and its impact on PET‐measured SUVs. J Nucl Med. 2004;45(8):1287–92. 15299050

[11] Vogel WV , van Dalen JA , Wiering B , et al. Evaluation of image registration in PET/CT of the liver and recommendations for optimized imaging. J Nucl Med. 2007;48(6):910–19. 17504865 10.2967/jnumed.107.041517

[12] Nehmeh SA and Erdi YE . Respiratory motion in positron emission tomography/computed tomography: a review. Semin Nucl Med. 2008;38(3):167–76. 18396177 10.1053/j.semnuclmed.2008.01.002

[13] Hamill JJ , Bosmans G , Dekker A . Respiratory‐gated CT as a tool for the simulation of breathing artifacts in PET and PET/CT. Med Phys. 2008;35(2):576–85. 18383679 10.1118/1.2829875

[14] Papathanassiou D , Becker S , Amir R , Menéroux B , Liehn JC . Respiratory motion artefact in the liver dome on FDG PET/CT: comparison of attenuation correction with CT and a caesium external source. Eur J Nucl Med Mol Imaging. 2005;32(12):1422–28. 16133387 10.1007/s00259-005-1868-y

[15] Goerres GW , Burger C , Kamel E , et al. Respiration‐induced attenuation artifact at PET/CT: technical considerations. Radiology. 2003;226(3):906–10. 12616024 10.1148/radiol.2263011732

[16] Beyer T , Antoch G , Blodgett T , Freudenberg LF , Akhurst T , Mueller S . Dual‐modality PET/CT imaging: the effect of respiratory motion on combined image quality in clinical oncology. Eur J Nucl Med Mol Imaging. 2003;30(4):588–96. 12582813 10.1007/s00259-002-1097-6

[17] Pan T , Mawlawi O , Nehmeh SA , et al. Attenuation correction of PET images with respiration‐averaged CT images in PET/CT. J Nucl Med. 2005;46(9):1481–87. 16157531

[18] Pan T , Mawlawi O , Luo D , et al. Attenuation correction of PET cardiac data with low‐dose average CT in PET/CT. Med Phys. 2006;33(10):3931–38. 17089855 10.1118/1.2349843

[19] Alessio AM , Kohlmyer S , Branch K , Chen G , Caldwell J , Kinahan P . Cine CT for attenuation correction in cardiac PET/CT. J Nucl Med. 2007;48(5):794–801. 17475969 10.2967/jnumed.106.035717 PMC2585486

[20] Liu C , Pierce LA 2nd , Alessio AM , Kinahan PE . The impact of respiratory motion on tumor quantification and delineation in static PET/CT imaging. Phys Med Biol. 2009;54(24):7345–62. 19926910 10.1088/0031-9155/54/24/007 PMC2895622

[21] Nehmeh SA , Erdi YE , Pan T , et al. Four‐dimensional (4D) PET/CT imaging of the thorax. Med Phys. 2004;31(12):3179–86. 15651600 10.1118/1.1809778

[22] Bundschuh RA , Martínez‐Moller A , Essler M , Nekolla SG , Ziegler SI , Schwaiger M . Local motion correction for lung tumours in PET/CT — first results. Eur J Nucl Med Mol Imaging. 2008;35(11):1981–88. 18682940 10.1007/s00259-008-0868-0

[23] Wolthaus JW , van Herk M , Muller SH , et al. Fusion of respiration‐correlated PET and CT scans: correlated lung tumour motion in anatomical and functional scans. Phys Med Biol. 2005;50(7):1569–83. 15798344 10.1088/0031-9155/50/7/017

[24] Park SJ , Ionascu D , Killoran J , et al. Evaluation of the combined effects of target size, respiratory motion and background activity on 3D and 4D PET/CT images. Phys Med Biol. 2008;53(13):3661–79. 18562782 10.1088/0031-9155/53/13/018

[25] Pevsner A , Nehmeh SA , Humm JL , Mageras GS , Erdi YE . Effect of motion on tracer activity determination in CT attenuation corrected PET images: a lung phantom study. Med Phys. 2005;32(7):2358–62. 10.1118/1.1943809 16121593

[26] Pönisch F , Richter C , Just U , Enghardt W . Attenuation correction of four dimensional (4D) PET using phase‐correlated 4D‐computed tomography. Phys Med Biol. 2008;53(13):N259–N268. 18562779 10.1088/0031-9155/53/13/N03

[27] Lujan AE , Balter JM , Ten Haken RK . A method for incorporating organ motion due to breathing into 3D dose calculations in the liver: sensitivity to variations in motion. Med Phys. 2003;30(10):2643–49. 14596301 10.1118/1.1609057

[28] Seppenwoolde Y , Shirato H , Kitamura K , et al. Precise and real‐time measurement of 3D tumor motion in lung due to breathing and heartbeat, measured during radiotherapy. Int J Radiat Oncol Biol Phys. 2002;53(4):822–34. 12095547 10.1016/s0360-3016(02)02803-1

